# The Impact of Coworkers’ Safety Violations on an Individual Worker: A Social Contagion Effect within the Construction Crew

**DOI:** 10.3390/ijerph15040773

**Published:** 2018-04-17

**Authors:** Huakang Liang, Ken-Yu Lin, Shoujian Zhang, Yikun Su

**Affiliations:** 1 School of Management, Harbin Institute of Technology, Harbin 150001, China; hkliang5493@hit.edu.cn; 2 Department of Construction Management, University of Washington, Seattle, WA 98195, USA; kenyulin@uw.edu; 3 School of Civil Engineering, Harbin Institute of Technology, Harbin 150090, China; 4 School of Civil Engineering, Northeast Forestry University, Harbin 150040, China; suyk@nefu.edu.cn

**Keywords:** social contagion, situational safety violations, routine safety violations, social learning, social information processing

## Abstract

This research developed and tested a model of the social contagion effect of coworkers’ safety violations on individual workers within construction crews. Both situational and routine safety violations were considered in this model. Empirical data were collected from 345 construction workers in China using a detailed questionnaire. The results showed that both types of safety violations made by coworkers were significantly related to individuals’ perceived social support and production pressure. Individuals’ attitudinal ambivalence toward safety compliance mediated the relationships between perceived social support and production pressure and both types of individuals’ safety violations. However, safety motivation only mediated the effects of perceived social support and production pressure on individuals’ situational safety violations. Further, this research supported the differences between situational and routine safety violations. Specifically, we found that individuals were more likely to imitate coworkers’ routine safety violations than their situational safety violations. Coworkers’ situational safety violations had an indirect effect on individuals’ situational safety violations mainly through perceived social support and safety motivation. By contrast, coworkers’ routine safety violations had an indirect effect on individuals’ routine safety violations mainly through perceived production pressure and attitudinal ambivalence. Finally, the theoretical and practical implications, research limitations, and future directions were discussed.

## 1. Introduction

Traditionally, numerous efforts to improve construction safety have been focused on improving site conditions, such as allocation of personal protection equipment, arrangement of physical work environment, and adoption of management systems [1,2,3]. Accordingly, construction environments have become dramatically safer over the past several decades [2]. Despite these improvements, worldwide, the construction industry still has a high frequency and severity of occupational injuries and fatalities [1,4,5], and much can still be done to improve construction safety [6]. According to Heinrich (1959), industrial accidents stem from the coincidence of unsafe behaviors and unsafe conditions [7]. Considering that nearly 80% of on-site accidents are caused by unsafe human behaviors, increasing understanding of, and eliminating other causes besides physical conditions—namely the unsafe behaviors of construction workers—is urgently needed [5].

From the results of previous behavioral investigations, we know that violations of safety rules and procedures are pervasive among construction workers [8]. For instance, Fang and Wu (2013) found that one third of construction workers did not follow the safety rules on two Singapore constriction sites [9], while Lipscomb et al. (2008) observed that construction workers often did not use fall prevention equipment [10]. Researchers have defined most violations as intentional but non-malevolent [11,12]. Here, “intentional” means that violations are distinct from human error, and are committed intentionally for various purposes such as saving time [1]. Human error is mainly derived from informational problems, where the information is forgotten, incomplete, incorrect, or unknown, while safety violations mainly involve motivational factors and are affected by social norm [13]. The term “non-malevolent” means that violators do not intend to cause accidents or damages to the system [14], which are different from the malevolent violations such as sabotage [11]. This is understandable because construction workers often encounter contradictory situations where they must balance conflicting work objectives, such as safety and production [15]. From this standpoint, coworkers’ intentional but non-malevolent violations can be practical and socially contagious because such deviants from formal systems or processes seem to be well-intentioned and aimed at getting the work done. Previous literature on workers’ safety behaviors or violations has focused on hierarchical supervisor-employee relationships, while the horizontal coworker-employee dynamics such as the contagion effect of coworkers’ safety violations have not received as much attention [16]. Therefore, it is necessary to examine the underlying mechanism of social contagion which is caused by coworkers in order to provide necessary insights into the processes by which unsafe behavior norms are established within a construction crew. These insights will shed light on how to prevent or reduce this type of misconduct from a new perceptive.

In this research, the “social contagion effect” refers to the process by which individuals adopt their coworkers’ attitudes, beliefs, or behaviors [17]. Coworkers have long been considered important sources of social influence in a group context [18,19,20,21]. According to Latane (1981) [22], social influence is the function of power, proximity, and number of referents exerting their influence. Coworkers tend to outnumber managers and supervisors, have closer proximity to fellow workers, and possess more field experience [23]. Thus, coworkers have more social influence on individuals. Previous studies have explored various aspects of the social contagion effect that coworkers have on individual workers, such as absenteeism [24], aggressive behavior [25], organizational citizenship behavior [26], and risk taking behavior (e.g., smoking, drug and alcohol use) [27]. This research focused on coworkers’ social influence within the construction industry, and specifically explored the social influence of coworkers’ safety violations on individual workers’ decisions to break the safety rules. 

Safety violations here are, to some extent, similar to “workarounds.” a concept that has been widely used in health care [28]. “Workarounds” means that, in complex socio-tech system, workers often work around the blocks or barriers in workflow to get job done [29]. The blocks or barriers have different forms based on intentions [30]. Some blocks are intended; they are intentionally included in a system as important controls to improve safety. For example, detailed safety check for personal protective equipment (PPE) is required before all activities onsite. By contrast, other blocks or barriers are unintentional, and exist in a system that are not anticipated in the design of the work procedure such as a lack of proper safety resources and adverse work environment. In this research, such unintentional blocks are labelled by “situational constraints.” Therefore, safety violations can be primarily categorized into routine and situational violations based on whether situational constraints are the main cause for violations. Routine violations occur when workers work around some safety procedures in order to realize organizational benefits (e.g., getting the job done in a timely manner) or personal gain (e.g., saving time or effort). When committing routine violations, workers habitually take the path of least effort or are “cutting corners” [31]. By contrast, situational violations tend to be driven by situational constraints in workflow, which make it difficult or impossible to follow the rules [1]. For example, workers may break rules when the PPEs are not readily available. Previous empirical research has found that situational and routine safety violations involve different antecedents [32,33]. For example, Hansez and Chmiel’s (2010) research found that routine safety violations were more associated with job demands, such as work overload [32]. By contrast, Chmiel et al.’s (2017) investigation argued that situational safety violations are predicted by how workers participate in non-mandatory safety activities [33]. Therefore, it is necessary to distinguish between these two types of safety violations and explore whether they have different social contagion processes. In the following section, we will develop a hypothesized model of social contagion effect based on a review of coworkers’ social influence and important variables. 

## 2. Literature Review

In the construction industry, work crews are spatially dispersed and have responsibility for various tasks away from the contractor’s office. The mobile workforce and pervasive subcontracting system further causes a loose connection between construction workers and top management [34]. Frontline workers are most likely to be influenced by their daily interactions with other members of their construction crews (e.g., the supervisor and coworkers) [35]. Previous studies have examined the impact of hierarchical supervisor-employee relationships on safety [36,37]. For instance, Shen et al. (2017) explored the impact of frontline supervisors’ transformational leadership on the safety behaviors of construction workers [36]. However, research on horizontal relationships with coworkers is still limited. Compared with the vertical leadership relationships with supervisors, coworkers’ attitudes and behaviors can provide more social information about what type of behaviors are socially accepted within a construction crew [23,38,39]. For instance, Tucker et al. (2008) reported that coworkers’ safety support exerted a greater influence on individual safety behavior than safety support from supervisors and senior management [40]. To this end, coworkers were selected as critical social referents in this research. As an important source of social influence, coworkers’ safety violations should have a dramatic influence on individual safety violations. 

Among various theoretical perspectives, social learning theory [41] and social information processing theory [42] are most commonly used to interpret the social contagion effect that coworkers have. Social learning theory describes how individuals use role models to learn about acceptable behaviors within a group [41]. Social information processing theory posits that individuals tend to search their social environment for information cues when they encounter ambiguous or uncertain aspects of their work, such as when there is a conflict between production and safety [17]. Both theories identify horizontal dynamics through which individuals are motivated to belong to their social group [24]. Both theoretical perspectives can be used to understand the social influence of coworkers’ safety violations. First, according to social learning theory [41], when coworkers are perceived to be accomplishing production tasks in an efficient manner (e.g., cutting-corners) they provide a model for individuals’ decisions about how to allocate available attention and effort between safety and production. Accordingly, when coworkers frequently break the safety rules, safety violations will be more acceptable to individual workers as a common strategy for addressing safety-production relationships. Second, according to social information processing theory, when encountering unstructured and ambiguous situations (e.g., safety-production conflicts), construction workers tend to reduce uncertainty by seeking information about acceptable behaviors from their coworkers [17]. Therefore, based on both social learning and social information processing theories, the following section reviews past literature and develops a model of the social contagion effect of coworkers’ safety violations within construction crews. 

### 2.1. Social Learning: Coworkers’ and Individuals’ Safety Violations

Social learning theory posits that any behaviors that can be learned via direct experience can also be learned by vicarious learning or role modelling, via observing other people’s behavior and associated consequences [41]. An individual can quickly reproduce modelled behavior via a successful imitating process [41]. Therefore, social learning can be viewed as a means through which values, attitudes, and behaviors are diffused in a social context [43]. The behavioral target of such processes could be safety violations within a construction crew. In this research, safety violations are defined as workers’ intentional deviations from safety rules to get job done, which are not meant to cause damages to the system [11]. From this standpoint, safety violations can be regarded as a special type of strategies or decision-making process to address complicated production processes. Individuals can thus observe and learn from the safety violations of their coworkers when they need to address the conflicting relationships between safety and production. According to social learning theory, effective role modelling requires that attention be focused on both the model and the modelled behavior [41]. Coworkers are front-line employees who have the same status and are faced with the same complicated work environment as the individuals. Coworkers are able to serve as appealing and credible role models because of their rich operational experience and proximity, which managers or supervisors do not necessarily have [44,45]. In addition, apprenticeship programs are common in the construction industry, where managers tend to pair-up experienced workers (mentors) with inexperienced or new workers [46,47]. During the apprenticeship period, inexperienced workers observe and learn more operational skills from their coworkers. Coworkers’ safety violations draw the attention of individuals because of the interdependence of their construction tasks, especially when facing intense time pressure or other challenges. In addition, a behavior’s consequences (rewards or punishments) also facilitate the modelling process. Through observation, an individual can learn the extent to which safety violations will be rewarded or punished in their organization. When they observe that the organization is lenient with safety violations, individuals are more likely to imitate such behaviors.

Drawing on social learning theory, previous studies have shown how coworkers influence individuals’ safety violations or compliance through role modelling. Westaby et al. (2005) discovered that risk taking behavior by coworkers is a significant predictor of young workers’ risk-taking orientation [48]. McLain (2014) found that coworkers are important social referents in relation to individuals perceptions of safety risks in highly hazardous work environments [49]. Gao et al. (2016) reported that coworkers as role models have great influence on construction workers’ safety performance because they provide beliefs about what kinds of behaviors are socially acceptable within the group [38]. Therefore, this research proposes that coworkers’ safety violations, both routine and situational, will foster such behavior by individuals. The corresponding hypotheses are as follows:

**Hypothesis** **1.** Coworkers’ situational safety violations will be positively related to individuals’ situational safety violations.

**Hypothesis** **2.** Coworkers’ routine safety violations will be positively related to individuals’ routine safety violations.

### 2.2. Social Information Processing: Perceived Production Pressure and Social Support

Beyond vicarious learning or role modelling appropriate work behaviors, social information processing theory posits that individuals seek social cues to help them interpret, and form attitudes about, uncertainty and ambiguity in their environment [42]. The social cognitions required to interpret social cues are critical in shaping workers’ personal attitudes [49]. As a subset of social cognitions, social safety cognitions refer to the mental operations which involve interpreting and perceiving others’ attitudes and behaviors for addressing safety issues [50]. Social safety cognitions mediate the relationship between safety cues, individuals’ safety attitudes, and behaviors [49,51]. In other words, this process begins with individuals seeking social cues (e.g., coworkers’ attitudes and behaviors) in order to interpret the ambiguous job characteristics such as the true priority of safety onsite. Then, individuals develop their own beliefs about what they should and should not do at work [21]. Production pressure (e.g., how the workers deal with the conflicts between safety and production) and safety-specific social support (e.g., sharing of information about hazards among workers) are the direct job characteristics that affect a worker’s safety behavior, motivation, and attitude [52]. According to the job demands–resources model [53], production pressure and social support represent two different processes by which workers react to their working conditions [32,54]. Production pressure is one salient job demand that requires sustained physical and psychological effort, and depletes energy, while social support, as a job resource, is a motivational process that is necessary to deal with job demand [55]. Therefore, in this study, two measures of social safety cognitions are incorporated: perceived production pressure and perceived social support.

Perceived production pressure—including excessive workload, required work pace, and time pressure—is frequently cited as a cause of safety violations and accidents [51,52,56,57,58]. Mullen (2004) reported that construction workers often operate in an unsafe manner not because they are unaware of the safety risks involved, but because of production pressure [59]. Production pressure is common on site because of the high interdependency of construction processes: a delay in one area can cause costly delays in others. Workers who perceive a high degree of production pressure will focus their attention on completing the work quickly, and will cut corners in order to satisfy their superiors and avoid negative production performance [52]. The safety violations investigated here are intentional but non-malevolent, which are committed for getting job done but not causing any harms to themselves or others. Hence, it is reasonable to suggest that coworkers who break safety rules with a well-intention to get job done can influence individual perceptions of production pressure, as coworkers are likely to provide cues that suggest work must be accomplished quickly and bending some safety rules is acceptable when the schedule is tight. When such safety violations are the norm, coworkers tend to value production over safety, and cut corners to achieve a higher production performance [1], which can eventually cause a greater production pressure for individuals. Even if individuals have legitimate reasons for not committing safety violations, co-workers and superiors might interpret these behaviors as uncooperative and unreliable [1]; an individual’s decision not to commit safety violations could negatively influence the groups’ production performance, due to the interdependence of construction tasks [47]. Therefore, this research proposes that:

**Hypothesis** **3a.** Coworkers’ situational safety violations will be positively related to individuals’ perceived production pressure.

**Hypothesis** **3b.** Coworkers’ routine safety violations will be positively related to individuals’ perceived production pressure.

Among the job resources included in the job demands–resources model, social support is considered to be one of the most important resources affecting construction workers’ safety-related behaviors [52,54]. Social support refers to the resources (emotional, functional, and informational) provided by members of the work environment, including management and coworkers, and the quality of these resources [60]. Safety-specific social support (henceforth shortened to “social support”) involves the necessary supports from others to avoid potential injuries or accidents, which can reveal how members of an organization value workers’ safety and safety issues in general [52]. Such support influences the balance of priorities between production and safety, and motivates individuals to perform their work in a safer manner [23]. However, when safety violations are the norm, coworkers who violate the safety rules are more likely to focus on production rather than safety, and are more reluctant to voluntarily engage in some proactive safety behaviors that are beneficial for other workers (e.g., sharing information on hazards with other workers) [61]. Thus, if their coworkers perform in an unsafe manner, individuals will have a negative perception of coworkers’ social support for safety. Furthermore, coworkers’ safety violations will be associated with lower levels of perceived management’s support, because they signal to individuals that the organization does not value safety enough and not provide a supportive work environment that would benefit the safety of workers. Based on this reasoning, the following hypotheses are proposed:

**Hypothesis** **4a.** Coworker’s situational safety violations will be negatively related to individuals’ perceived social support.

**Hypothesis** **4b.** Coworker’s routine safety violations will be positively related to individuals’ perceived social support.

### 2.3. Social Information Processing: Attitudinal Ambivalence and Safety Motivation

Drawing on social information processing theory, job attitudes of workers are mainly derived from the information processing in their social environment [42]. As opposed to traditional univalent safety attitudes (i.e., predominantly positive or negative), attitudinal ambivalence is more consistent with the psychological conflict when an individual decides to violate on-site safety rules. Attitudinal ambivalence refers to the extent to which an individual simultaneously holds both positive and negative attitudes toward an object or behavior [62]. Attitudinal ambivalence is often an individual’s reaction to conflicting cues, and serves as an adaptive function in a social environment filled with pressures [63]. Safety violations are often the results of such conflicting cues due to the contradiction between perceived benefits and costs [15]. In other words, workers know safety violations are risky, but they cannot stop such behaviors because of the associated increased production and income. Prior research has examined the attitudinal ambivalence aroused by work-related safety behavior. For instance, Cavazza and Serpe (2009) measured workers’ attitudinal ambivalence toward using PPEs [15]. Their research found that an organization’s safety climate is negatively related to attitudinal ambivalence toward safety compliance, which is in turn positively related to the safety violations of workers. According to social information processing theory, the social environment (e.g., behaviors and attitudes of others) can provide such conflicting information about the safety-production relationship, which could facilitate individuals’ attitudinal ambivalence. Individuals with high attitudinal ambivalence are receptive to opposing views of organizational safety procedures, and are more likely to violate safety rules because they have not yet established firm beliefs regarding safety behaviors [64]. 

As mentioned above, perceived social support and production pressure, two critical social safety cognitions, contribute to individuals’ attitudinal ambivalence toward safety compliance. Specifically, social support is expected to reduce individuals’ attitudinal ambivalence toward safety compliance, since higher support for safe behaviors might decrease the perceived costs of safety compliance (e.g., assistance from coworkers could decrease the time needed to wear safety equipment), and increase the perceived benefits (i.e., potential rewards for safety from the organization). By contrast, perceived production pressure might increase individuals’ attitudinal ambivalence toward safety compliance because workers must temporarily prioritize production in situations of extreme time pressure. Accordingly, this research proposes that attitudinal ambivalence will mediate the effect of social safety cognitions on individuals’ safety violations. Specifically, when perceiving more production pressure, individuals will have higher attitudinal ambivalence, while when perceiving more social support, they will have lower attitudinal ambivalence. Then, attitudinal ambivalence will be positively related to individuals’ situational and routine safety violations. Therefore, the following hypotheses are formulated:

**Hypothesis** **5a.** Perceived social support will be negatively related to attitudinal ambivalence toward safety compliance.

**Hypothesis** **5b.** Perceived production pressure will be positively related to attitudinal ambivalence toward safety compliance.

**Hypothesis** **6a.** Attitudinal ambivalence toward safety compliance will mediate the relationships between perceived social support and both routine and situational safety violations committed by individual workers.

**Hypothesis** **6b.** Attitudinal ambivalence toward safety compliance will mediate the relationships between perceived production pressure and both routine and situational safety violations committed by individual workers.

As mentioned above, safety violations are mainly intentional but non-malevolent [11,12], and are caused by some motivational factors, such as lack of safety motivation [13]. Safety motivation is regarded as a critical proximal determinant of safety violation or compliance, which has long been established in previous research (e.g., [52,65,66]). Individuals tend to adapt their safety motivation to their social environment, where the more violations their coworkers are perceived to commit, the lower an individual’s motivation to work safely. Like attitudinal ambivalence, perceived social support and production pressure are expected to shape individuals’ safety motivation, which in turn affects safety violations [52]. Perceived social support will likely motivate individuals to behave more safely, while perceived production pressure will likely decrease individuals’ safety motivation. Accordingly, this research proposes that safety motivation will meditate the relationships between perceived production pressure and social support, and individuals’ safety violations. The hypotheses are formulated as follows:

**Hypothesis** **7a.** Perceived social support will be positively related to individuals’ safety motivation.

**Hypothesis** **7b.** Perceived production pressure will be negatively related to individuals’ safety motivation.

**Hypothesis** **8a.** Safety motivation will mediate the relationships between perceived social support and both routine and situational safety violations committed by individual workers.

**Hypothesis** **8b.** Safety motivation will mediate the relationships between perceived production pressure and both routine and situational safety violations committed by individual workers.

**Figure 1:**
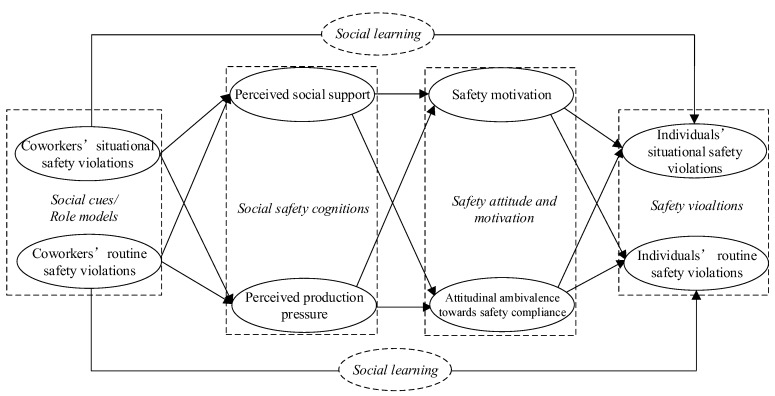
This study’s hypothesized model.

Our hypothesized theoretical model is summarized in Figure 1. This model posits that the direct effect of coworkers’ safety violations (i.e., role models) on individual workers’ safety violations takes place through the mechanism of social learning. Our model also presents the indirect effects of coworkers’ safety violations (i.e., social cues) as taking place through social information processing: coworkers have indirect effects on the safety violations of individual workers via social safety cognitions (variables are perceived social support and production pressure) and safety motivation and attitudes (variables are safety motivation and attitudinal ambivalence toward safety compliance). 

## 3. Method 

### 3.1. Measures 

#### 3.1.1. Coworkers and Individuals’ Safety Violations

The questions for assessing both coworkers and individuals’ safety violations were adapted from the self-report items validated by Hansez and Chmiel (2010) [32] and Chmiel et al. (2017) [33]. Situational safety violations were measured with six items and were inversely scored: a high score indicated a low level of violations. A sample item is “My coworkers always use safety equipment, even when it’s not easily available.” Routine violations were measured with four items, and a high score indicated a high level of violations. A sample item is “My coworkers sometimes cut corners if it makes the task easier.” In measuring individuals’ situational and routine safety violations, “My coworkers” was replaced in all assessment items such that the individual worker was the subject. Accordingly, the above-mentioned sample items were expressed as “I always use safety equipment, even when it’s not easily available” and “I sometimes cut corners if it makes the task easier.” To avoid any misunderstanding among respondents, coworkers’ safety violations were measured at the beginning of the questionnaire, and individual safety violations were measured at the end. Respondents were also informed of the difference between two targeted subjects before they responded to the questionnaires. 

#### 3.1.2. Perceived Social Support and Production Pressure 

Drawing on the hypothesized social contagion model in Figure 1, social safety conditions were assessed by asking questions regarding perceived social support and production pressure [54]. Perceived social support was measured with four items that described the safety-specific support received from the organization and coworkers [52,67]. Sample items include “Management can always deal with the safety issues reported by workers in a timely manner” and “There is frequent communication about safety issues within our workgroup.” Perceived production pressure was measured with six items from previous research [52,57,68]. Sample items are “Sometimes there is not enough time available for following safety rules and procedures” and “Short cuts and risk taking are common due to the heavy workload.” 

#### 3.1.3. Safety Motivation and Attitudinal Ambivalence Toward Safety Compliance

Safety motivation was assessed by using another four items, which were constructed based on previous studies [52,68,69]. These items measured individual motivation to perform safety-related activities. Sample items include “I feel guilty when I don’t work safely,” and “I feel that it is worthwhile to put in effort to maintain or improve workplace safety.” According to Cavazza and Serpe (2009) [15], four items can be used to measure individuals’ attitudinal ambivalence toward safety. Positive and negative attitudes toward safety compliance were assessed separately. The four items include “Following safety procedures makes working more difficult,” “Wearing personal protective equipment bothers my daily work,” “Wearing personal protective equipment helps me avoid possible damage,” and “Following safety procedures makes me feel safe.” Finally, a global ambivalence score was calculated using the formula suggested by Ran and Yamamoto (2015) [64]. Specifically, the average of the positive and negative attitude scores was determined, and then the absolute difference between the two components was subtracted from the average (as shown in Equation (1)). P denotes the total positive attitude score, and N denotes the total negative attitude score. Higher scores indicate a greater level of ambivalence.

(1) Attitudinal ambivalence=(P+N)/2−|P−N|

### 3.2. Participants and Questionnaire Administration

Before the formal investigation, a pilot study was conducted to ensure that the intended questions were applicable given that some measurements were borrowed from previous studies of other industries. A total of 19 workers participated in this pilot, based on which the initial questionnaire was revised. For example, some questions were rephrased so they would be clearer to workers with a limited education. The final questionnaire consisted of two parts: general demographic questions and safety-specific questions. Five demographic questions asked about respondents’ gender, age, years of work experience, education level, and trade type. Thirty-eight safety-specific questions were evaluated on a five-point Likert scale ranging from one (strongly disagree) to five (strongly agree). Safety-specific questions were developed with the aim of collecting meaningful data that could capture the mediating effect of the factors hypothesized in Figure 1. The complete items for all variables have been provided in Supplementary Materials.

The formal questionnaires were administrated to construction workers in four high-rise residential construction projects located in three different Chinese cities (two in Harbin, one in Shenyang, and one in Zhengzhou). The contractors involved in the four projects are all large government-owned enterprises. After obtaining permission from the senior management, in August and September 2017, the questionnaire was distributed in person to a total of 550 construction workers. The workers were assured of anonymity and not required to provide any personal or identifiable information in the questionnaire. In addition, they were free to decline to participate, without consequence, at any time prior to or at any time point during this investigation. The questionnaire was primarily conducted at the jobsite during work hours, which unavoidably interrupted ongoing work. To encourage participation, the researchers communicated the pure academic purposes with the immediate management of workers to get them on board. Meanwhile, monetary compensation was provided to workers who participated in the research and answered all the questions. For workers who could not fully understand the questionnaire, the researchers helped them complete it. Finally, after removing incomplete responses, a total of 345 valid responses were used in this study (a response rate of 62.7%). 

Table 1 summarizes respondents’ demographic characteristics. Respondents were mostly male (94.2%) due to the male-dominant workforce in the Chinese construction industry. Among the respondents, those belonging to the age groups of 30–39 and 40–49 accounted for the largest proportion (37.7% and 35.7%, respectively). A total of 16.8% of respondents had less than five years of work experience in construction, and 66.4% had 6–15 years of experience. The majority of respondents (72.2%) had completed primary or junior high school as their highest education, indicating that the level of education received by Chinese construction workers is still far from ideal [70]. Respondents were primarily from eight trade types: general (11.0%), steel (11.0%), concrete (4.9%), scaffolding (6.7%), carpenter (22.9%), plasterer (10.4%), bricklayer (8.1%), and welding (8.7%). 

### 3.3. Statistical Procedures

Based on the data collected, the hypotheses regarding the social contagion process of safety violations were tested using the structural equation modeling (SEM) technique. Specifically, a two-stage SEM approach was carried out to verify the measurements and structural models. First, confirmatory factor analysis (CFA) was conducted to verify the reliability and validity of the measurement model. Next, the structural model was estimated by using path analysis to test the hypotheses, especially the relationships among different latent constructs. At present, there is still no consensus concerning the best indices for assessing the overall fitness of SEM models [71]. In current research, a number of commonly used indices were used to assess the model fit. These indices included the ratio of model chi-square to the degrees of freedom ($\frac{\chi^{2}}{df}$), the root mean square error of approximation (RMSEA), the Incremental Fit index (IFI), the Tucker-Lewis index (TLI), and the Comparative Fit index (CFI) [52]. Values of $\frac{\chi^{2}}{df}$ of 3.0 or less, RMSEA of 0.08 or less, CFI, TLI and IFI of 0.90 or higher, suggest a good model fitness [36,38,52]. Analysis of Moment Structures (AMOS) v21.0 and SPSS v18.0 (IBM, Armonk, New York, USA) were used to process the empirical data and perform statistical analyses. 

## 4. Analysis and Results 

### 4.1. Measurement Model Assessment

The measurement model assessment is concerned with how well the items measure the latent constructs according to their reliability and validity. In the reliability test, Cronbach’s α was used to assess internal consistency reliability, which reflects correlations between measurement items belonging to one dimension [72]. A Cronbach’s α value above 0.70 is recommended to ensure the data reliability [73]. Next, two types of commonly reported construct validity, namely convergent and discriminant validity, were tested [36,52]. Convergent validity assesses the degree to which a latent construct’s question items are highly correlated, while discriminant validity measures the extent to which one construct is distinct from others. Convergent validity can be verified using standardized factor loading (FL), composite reliability (CR), and average variance extracted (AVE). A standardized FL value of 0.70 or higher [74], CR of 0.70 or higher [75], and AVE of 0.50 or higher [36,38] suggest adequate convergence. In addition, the square root of AVE values should be higher than their inter-construct correlations to achieve discriminant validity [76]. 

**Figure 2:**
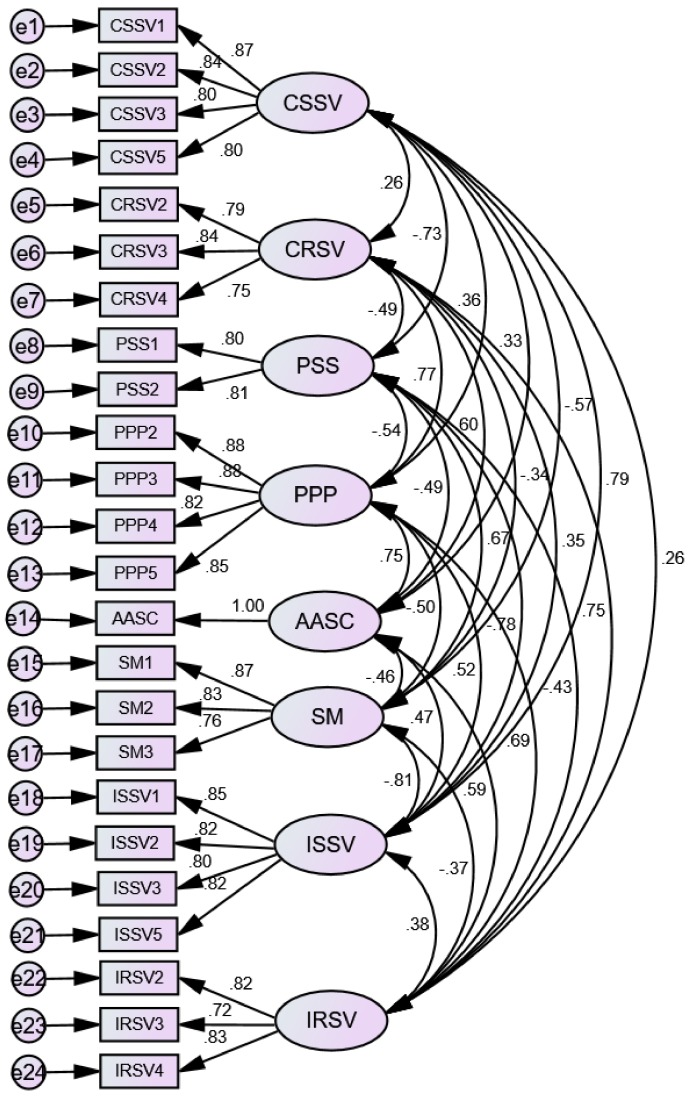
Final measurement model generated by AMOS v21 ($\frac{\chi^{2}}{df}\;$= 1.752; CFI = 0.971; TLI = 0.964; IFI = 0.971; RMSEA = 0.047).

To adapt to the software environment, some abbreviations were used in the statistical analyses to represent the eight constructs used (shown in Table 2). According to the suggestion made by Wu (2009) [74], the items with low FL values were dropped in the confirmatory factor analysis. The final measurement model with acceptable goodness-of-fit is shown in Figure 2 ($\frac{\chi^{2}}{df}\;$= 1.752; CFI = 0.971; TLI = 0.964; IFI = 0.971; RMSEA = 0.047). All FL values of measurement items were statistically significant at the 0.001 level. Table 3 shows the results of the reliability and convergent validity assessment, including the values of Cronbach’s α, CRs, and AVEs. The Cronbach’s α values were all greater than 0.70, which confirms strong internal consistency reliability of the constructs. The AVEs and CRs of all constructs satisfied the threshold values of AVE of 0.5 and CR of 0.70, providing evidence of adequate construct convergence. The discriminant validity of all constructs was verified because the square roots of AVEs of any two constructs (bold figures in Table 4) were greater than their inter-construct correlations. As shown in Table 4, all constructs were significantly correlated (*p* < 0.001). None of the correlation values exceeded the threshold value of 0.90, suggesting the absence of multi-collinearity [75]. 

### 4.2. Structural Model Assessment

**Figure 3:**
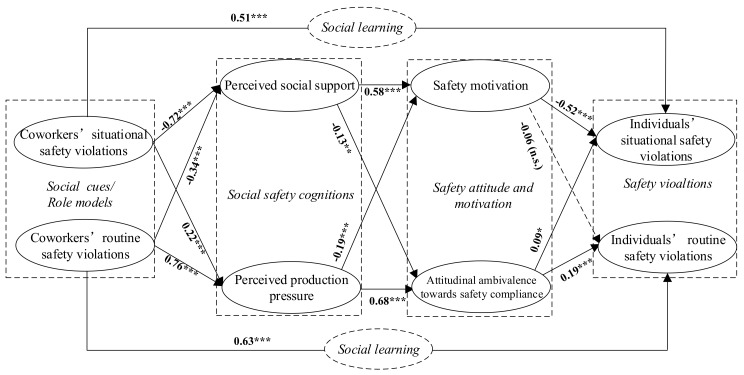
The estimated structural model for total sample of workers ($\frac{\chi^{2}}{df}\;$= 1.832; CFI = 0.965; TLI = 0.960; IFI = 0.966; RMSEA = 0.049). Note. *** *p* < 0.001, ** *p* < 0.01, * *p* < 0.05, n.s. *p* > 0.05.

After establishing the measurement model, the hypothesized structural model shown in Figure 1 was estimated. Figure 3 presents the estimated structural model and standardized path coefficients. The values of fit indices ($\frac{\chi^{2}}{df}\;$= 1.832; CFI = 0.965; TLI = 0.960; IFI = 0.966; RMSEA = 0.049) were all within the recommended limits, suggesting that the hypothesized model could adequately fit the empirical data. 

In the estimated structural model (Figure 3), if the standardized path coefficient is significant and in the hypothesized direction, then the corresponding hypothesis can be verified [36]. Specifically, Hypotheses H1 and H2 proposed that both types of coworkers’ safety violations would have positive relationships with their corresponding individuals’ safety violations. Both hypotheses were supported, and the results suggested coworkers’ routine safety violations (standardized path coefficient β=0.63; *p* < 0.001) had a stronger effect than situational safety violations (β=0.51; *p* < 0.001). Hypotheses H3 and H4 proposed that coworkers’ safety violations would predict two social safety cognitions: perceived social support and perceived production pressure. Four paths between coworkers’ safety violations and social safety cognitions were proven to have strong statistical significance, and thus hypotheses H3 and H4 were supported. Next, hypotheses H5 and H7 proposed that perceived social support and production pressure were related to safety motivation and attitudinal ambivalence toward safety compliance. Both hypotheses were supported because the four corresponding paths were all statistically significant. Further, hypothesis H6 proposed that attitudinal ambivalence would mediate the relationships between both types of social safety cognitions and individuals’ safety violations. This hypothesis was supported because, in addition to the significant paths from both types of social safety cognitions to attitudinal ambivalence as mentioned above, two paths from attitudinal ambivalence to both types of individuals’ safety violations were also significant. However, hypothesis H8 could not be verified completely because safety motivation had a significantly negative effect on individuals’ situational safety violations, but the direct path to individuals’ routine safety violations was not significant. Hence, safety motivation only mediated the relationships between social safety cognitions and individuals’ situational safety violations. 

## 5. Discussion

The results of this research support a theoretical model of safety violations’ social contagion effect within construction crews. This research extends previous research by suggesting that coworkers are critical social referents for shaping group safety norms [19,40,77]. This research treated coworkers’ safety violations as target behaviors, which are related to an individual’s inclination to violate safety rules through social learning and social information processing. This is the first research to explain the formation of unsafe group norms by using these two theoretical perspectives. Moreover, this research suggests that it is critical to recognize how different types of safety violations (e.g., situational and routine) can have different social contagion processes. To the best of our knowledge, this research is one of the few to empirically explore the distinctions among different types of safety violations. The findings of this research provide insight into how and why coworkers’ safety violations encourage individuals to commit safety violations within construction crews. 

### 5.1. The Discussion of General Findings

#### 5.1.1. The Influence of Coworkers’ Safety Violations on Individuals’ Safety Violations

Through hypotheses H1 and H2, this research found that coworkers’ safety violations were significantly related to individuals’ safety violations. This indicates that individuals who perceive higher levels of coworker safety violations are more likely to break safety rules. According to Bandura’s (1977) social learning theory, individuals learn acceptable and normative behaviors by observing how their coworkers behave in groups [41]. Coworkers often serve as critical on-site role models due to the interdependent nature of construction tasks, and especially considering the apprenticeship system in the construction industry [47]. Compared with management and supervisors, coworkers know more about the constantly changing work environment and their behaviors often provide strong and immediate references when there is tension between different job requirements such as production and safety [19]. As defined above, safety violations addressed in this research were intentional but non-malevolent, where workers know the safety rules, deviate from them, but do not desire to cause harm [12]. Management’s attitudes toward such violations may be ambiguous because such violations do not always cause injuries or illnesses and sometimes even seem unavoidable. Accordingly, when coworkers intentionally break some safety rules, individuals can observe these behaviors directly and learn how to operate in similar situations.

#### 5.1.2. The Influence of Coworkers’ Safety Violations on Social Safety Cognitions

Through hypotheses H3 and H4, this research found that both types of coworkers’ safety violations had significant relationships with perceived social support and production pressure. The results were consistent with the argument put forward in McLain and David’s (2014) model, where social cues in the work environment can trigger social safety cognitions (e.g., assessment of safety climate) [49]. This research used perceived social support and production pressure as its two social safety cognitions because they are critical aspects of job resources and demands, respectively [52,54]. These findings support the notion that individuals obtain information about job characteristics from their coworkers’ behaviors, and subsequently form their perceptions of the organization or its top management [42]. As hypothesized, the results showed that individuals who observed a greater number of coworkers’ safety violations had higher perceptions of production pressure, and lower perceptions of social support. These findings suggest that production-safety conflict and lack of necessary safety support may be the main reasons that construction workers break safety rules. The results are consistent with Wang’s (2013) survey on factors associated with workers’ safety violations in the Chinese construction industry [78]. In his survey, 96% of respondents agreed that there was a constant pressure to work at a high speed to complete projects on time [78]. This high production pressure may be partly due to increased demand to complete large-scale construction projects more quickly caused by the rapid pace of development currently taking place in China [78]. Social support from management and other group members is also critical because social support aids task completion and reduces the influences of other job demands such as time pressure [79]. 

#### 5.1.3. The Mediating Role of Attitudinal Ambivalence and Safety Motivation

Regarding hypotheses H5 and H6, this research found that individuals’ attitudinal ambivalence toward safety compliance mediated the relationships between their perceived social support and production pressure, and both types of safety violations. The findings are consistent with Cavazza and Serpe’s (2009) empirical research for the general industry, which found that workers’ attitudinal ambivalence mediated the relationship between a workplace’s safety climate and safety violations [15]. The results support the argument that construction workers tend to hold conflicting attitudes toward safety compliance. This research suggests that greater perceived production pressure increases the extent of individuals’ attitudinal ambivalence, while greater perceived social support decreases attitudinal ambivalence. Furthermore, individuals with high attitudinal ambivalence commit more safety violations. Through hypothesis H7, this research also found that both perceived production pressure and social support had significant relationships with safety motivation. These results are consistent with Guo et al.’s (2016) study on the influence of the workplace safety climate on construction workers’ safety behaviors in New Zealand [52]. Furthermore, safety motivation was found to be significantly related to individuals’ situational safety violations. However, the relationship between safety motivation and individuals’ routine safety violations was not statistically significant. This suggests that situational safety violations derive more from motivational problems than routine safety violations. 

### 5.2. The Distinctions between Situational and Routine Safety Violations

This research extends the scope of previous literature by considering the distinctions between different types of safety violations (situational and routine). The magnitude of the direct effect of coworkers’ routine safety violations on individuals’ routine safety violations (β=0.63; *p* < 0.001) was stronger than the direct effect of situational safety violations (β=0.51; *p* < 0.001). This finding suggests that individuals are more likely to observe and imitate coworkers’ routine safety violations than situational safety violations. Routine safety violations exert more social influence because they tend to be more common on site than situational violations, and they are often committed by experienced workers [80]. According to the magnitude of path coefficients shown in Figure 3, coworkers’ situational safety violations had an indirect effect on individuals’ safety violations mainly through perceived social support and safety motivation. On the other hand, coworkers’ routine safety violations had an indirect effect on individuals’ routine safety violations mainly through perceived production pressure and attitudinal ambivalence. These findings support and reinforce the notion that situational and routine safety violations are different types of violations with different mediators [32,33]. 

As mentioned above, situational safety violations are mainly caused by situational constraints [11]. Thus, social support (e.g., the extent to which management values workers taking initiative regarding safety) that may reduce such constraints should be more associated with situational violations. Moreover, a socially supportive work environment will motivate individuals to be proactive in promoting workplace safety (e.g., assisting others to obtain safety equipment in a timely manner when it is needed temporarily), which will eventually reduce situational safety violations. These findings support the assertion made by Chmiel et al. (2017) that situational safety violations are strongly associated with psycho-social mechanisms [33]. Their research found that workers’ participation in non-mandatory safety activities led to a decrease in situational safety violations [33]. It is worth to note that production pressure is also significantly related to situational safety violations (shown in Figure 3). For instance, when workers feel the time pressure, they are more inclined to break the safety rules if their PPEs are not readily available. However, the root cause for such situational safety violation is derived from situational constraints (i.e., lack of PPEs) rather than the limitation of time. Hence, the result of this research can be beneficial for mangers to make appropriate controls for different types of safety violations. 

By contrast, routine safety violations are associated with individuals’ cognitive energy or “effort,” as workers commit such violations by completing tasks using the least possible effort [32]. Thus, job demands, such as production pressure, that may deplete individuals’ energy or efforts, should have a greater influence on routine safety violations. Depletion of energy or efforts can further increase individuals’ attitudinal ambivalence toward safety compliance because of the effort and time-consuming processes required for safety compliance. This eventually leads to an increase in routine safety violations. The results of this research extend Hansez and Chmiel’s (2010) finding that job strain—a variable indicating the depletion of cognitive energy—causes routine safety violations, because they did not explore the mediating effect of attitudinal ambivalence toward safety compliance [32]. A better understanding of attitudinal ambivalence’s mediating effects provides helpful hints on the research and has practical implications. 

### 5.3. Theoretical Implications

The findings of this research have theoretical implications for both workplace social influence and occupational safety research. First, this research incorporates the social learning and social information processing theories to explain the social contagion effect of coworkers’ safety violations. This research reveals the horizontal coworker-employee dynamics, which complements the previous occupational safety research that mainly focused on the hierarchical supervisor-employee relationships such as safety leadership [36]. Second, the intentional but non-malevolent safety violations, and other similar behaviors, such as “workarounds” [81] and “cutting corners” [82], are common in many hazardous occupations where workers should deal with the conflicting relationships between safety and other production performance. This research gives the evidence that a group-level focus is appropriate and important for understanding safety violations because individuals’ decisions to break safety rules are closely related to their coworkers’ safety violations. Therefore, this research can provide some meaningful insights to safety-violations specific studies, and to other occupational-violations studies in general. In addition, this research should also apply to the members of other hazardous occupations such as healthcare and firefighting. Third, our findings provide empirical evidence that situational and routine safety violations involve different social contagion processes, extending previous literature that treated them as a single behavior. Finally, this research introduces some mediators, such as attitudinal ambivalence, which potentially provide a meaningful answer to the question of why individuals imitate coworkers’ safety violations. 

### 5.4. Practical Implications

In addition to the theoretical implications, the findings in this research also have several practical implications. Although organizational safety rules are essential for on-site safety, carrying out tasks with coworkers who value and prioritize safety should be just as important [77]. This research found that individuals who perceived a higher level of coworker safety violations were more prone to committing safety violations. Therefore, managers should not only focus on the possible explicit costs of safety violations (e.g., injuries or illnesses), but also on their social contagion effect. According to Heinrich’s safety pyramid theory [7], the accumulation of a large number of safety violations may eventually cause serious accidents. Therefore, managers should identify and eliminate safety violations timely before they spread throughout a construction crew via a social contagion effect. In addition to eliminating safety violations via direct supervision, managers can also reward safety role models: workers who are experienced and demonstrate good safety performance. Frequent communication and safety training can be strategically provided to these role models to ensure that they understand the organizational safety rules and have the capacity to balance conflicting work objectives. Through the placement of safety role models, organizations can exert continuous influence on workers’ safety behaviors. Further, managers should take different measures to prevent situational and routine safety violations. Specifically, managers should create a supportive environment by demonstrating that they care about the safety issues experienced by workers, and by emphasizing the importance of mutual safety support among workers. Such an environment can motivate workers to be proactive in promoting safety and can reduce situational safety violations. In addition, managers can help create a safe workplace by better sequencing work activities in order to avoid heavy production pressure. Finally, managers should improve the quality of safety equipment and the practicality of safety rules in order to avoid workers having attitudinal ambivalence toward safety compliance. Taking the above actions will eventually reduce routine safety violations. 

### 5.5. Research Limitations and Future Directions 

Despite the theoretical and practical contributions, this research had several limitations. First, this study’s cross-sectional design precluded the causal inferences. For instance, the hypothesized causal relationships between coworkers’ safety violations and perceived social support could function in opposite ways. A longitudinal or experimental design is needed to provide meaningful explanations in future studies. Second, although participation was anonymous by nature, workers tended to be worried that their safety violations would be exposed to their managers as a result of this research. The self-reported data might have reduced the accuracy of safety violation measures considering how workers might underreport their safety violations. Nevertheless, such possible bias is less of an issue in our research because we were not interested in explaining the absolute levels of safety violations. In addition, the intentional but non-malevolent nature of safety violations investigated possibly reduced the sensitivity of the subject. Furthermore, we have attempted to reduce underreporting by communicating the pure academic purpose with workers. Third, self-reported data can be especially problematic because it increases the possibility of common method variance (CMV). However, such common method variance was minimal in this research because the measurement model had good discriminant validity, and there was also a nonsignificant link in the final structural model, despite a large sample size [83]. The CMV problem could be further verified by comparing a 1-factor (i.e., all items loaded on a common factor) model to the 8-factor model [84]. The confirmatory factor analysis showed that the proposed 8-factor model provided a significantly better fit ($\frac{\chi^{2}}{df}\;$= 1.752; CFI = 0.971; TLI = 0.964; IFI = 0.971; RMSEA = 0.047) than the 1-factor model ($\frac{\chi^{2}}{df}$ = 15.070; CFI = 0.276; TLI = 0.221; IFI = 0.279; RMSEA = 0.202). Therefore, the results suggest that the issue of common method variance did not significantly influence our findings. Nonetheless, multisource data from supervisor- or coworker-reported and self-reported measures is suggested for future studies. 

The participants in this research were drawn from four construction projects in China, a country that is considered to have a collectivist culture. However, it is unclear whether the findings would hold in countries with individualistic cultures, such as the United States. Hence, subsequent studies should verify the current findings in diverse cultural and industrial settings. In addition, the suggested social learning mechanism could be further explored by examining whether vicarious learning—a variable indicative of the social learning process [41]—mediates the relationships between coworkers and individuals’ safety violations. Future research is also needed to further explore whether age [85], gender [12], and other personal traits such as “machismo” [86] can influence the social contagion effect of safety violations among construction workers. Other potential influential factors can be the close supervision by managers and the likelihood of punishment in the work environment, and the risk level of safety violations.

## 6. Conclusions

To the best of our knowledge, this research was the first to investigate the social contagion effect of safety violations. Safety violations are not simply an individual-level phenomenon, but also a social phenomenon. As the results of this research showed, individuals’ decisions to break safety rules are encouraged by their coworkers’ safety violations. Specifically, coworkers’ safety violations have a direct effect on individuals’ behavior through the observation learning mechanism. Meanwhile, coworkers also provide a social context (e.g., social cues) and have indirect effects on individuals’ safety violations via individuals’ interpretation of organization-level systems (e.g., perceived social support and production pressure), and individuals’ safety motivation and attitudinal ambivalence. These findings provide meaningful insights for understanding how horizontal group dynamics shape safety behaviors. To this end, this research adds a new perceptive to existing safety behavior literature, which has tended to mainly focus on hierarchical supervisor–employee relationship. Furthermore, our findings indicate the distinctions between situational and routine safety violations. According to the results, routine safety violations are more contagious than situational safety violations. The social contagion of situational safety violations was mainly mediated by perceived social support and safety motivation, while routine safety violations spread mainly through the mechanisms of perceived production pressure and attitudinal ambivalence. Finally, this research provides practical measures for managers to control the social contagion of different types of safety violations.

## Figures and Tables

**Table 1:** Demographic characteristics of respondents.

Characteristics	Category	Frequency (*N* = 345)	Percentage
Gender	Male	325	94.2%
	Female	20	5.8%
Age (years)	<20	1	0.3%
	20–29	61	17.7%
	30–39	130	37.7%
	40–49	123	35.7%
	≥50	30	8.7%
Work experience (years)	≤5	58	16.8%
	6–10	132	38.3%
	11–15	97	28.1%
	16–20	30	8.7%
	>20	28	8.1%
Highest level of education attained	Primary school	60	17.4%
	Junior high school	189	54.8%
	Senior high school	77	22.3%
	Vocational college	14	4.0%
	Bachelor degree and above	5	1.4%
Trades	General	38	11.0%
	Steel	38	11.0%
	Concrete	17	4.9%
	Scaffolding	23	6.7%
	Carpenter	79	22.9%
	Plasterer	36	10.4%
	Bricklayer	28	8.1%
	Welding	30	8.7%
	other	56	16.2%

**Table 2:** Glossary of abbreviations.

Abbreviations	Constructs
CSSV	Coworkers’ situational safety violations
CRSV	Coworkers’ routine safety violations
PSS	Perceived social support
PPP	Perceived production pressure
AASC	Attitudinal ambivalence toward safety compliance
SM	Safety motivation
ISSV	Individuals’ situational safety violations
IRSV	Individuals’ routine safety violations

**Table 3:** Descriptive statistics, construct reliability, and convergent validity.

Constructs	M	SD	Cronbach’s α	CR	AVE
CSSV	1.963	0.838	0.895	0.897	0.686
CRSV	2.465	1.006	0.834	0.836	0.630
PSS	4.133	0.750	0.788	0.786	0.656
PPP	2.420	0.888	0.919	0.918	0.736
AASC	2.990	3.093	-	-	-
SM	3.159	0.552	0.852	0.861	0.674
ISSV	1.912	0.783	0.894	0.893	0.677
IRSV	2.436	0.988	0.833	0.834	0.627

(1) Abbreviations: M = Mean; SD = Standard deviation; CR = Composite reliability; AVE = Average variance extracted. (2) Note: AASC is a one-indicator construct for which Cronbach’s Alpha, CR, and AVE were not assessed here.

**Table 4:** The results of discriminant validity**.**

No.	Constructs	1	2	3	4	5	6	7	8
1.	CSSV	0.828							
2.	CRSV	0.263 ***	0.794						
3.	PSS	−0.732 ***	−0.491 ***	0.810					
4.	PPP	0.364 ***	0.775 ***	−0.536 ***	0.858				
5.	AASC	0.327 ***	0.599 ***	−0.491 ***	0.748 ***	-			
6.	SM	−0.570 ***	−0.341 ***	0.670 ***	−0.499 ***	−0.456 ***	0.821		
7.	ISSV	0.790 ***	0.349 ***	−0.784 ***	0.523 ***	0.470 ***	−0.812 ***	0.823	
8.	IRSV	0.256 ***	0.750 ***	−0.427 ***	0.694 ***	0.594 ***	−0.369 ***	0.385 ***	0.792

(1) Correlations are below the diagonal, and the figures in bold on the diagonal are the square root of the average variance extracted (AVE) of associated constructs. (2) *** = Correlation is significant at the 0.001 level.

## Supplementary Materials

The following are available online at http://www.mdpi.com/1660-4601/15/4/773/s1
