# Supplementary material for: The Impact of Coworkers’ Safety Violations on an Individual Worker: A Social Contagion Effect within the Construction Crew
Source: Int J Environ Res Public Health. 2018 Apr 17;15(4):773. doi: 10.3390/ijerph15040773 (PMC5923815; doi:10.3390/ijerph15040773)
Supplement: Supplementary file 1 [file ijerph-15-00773-s001.pdf]

1. The 6 items of coworkers' situational safety violations

CSSV1: My coworkers always carry out their work in a safe manner.

CSSV2: My coworkers always report all safety-related incidents.

CSSV3: My coworkers always wear personal protective equipment, even when it's inconvenient and uncomfortable.

CSSV4: My coworkers never find following safety procedures a hassle.

CSSV5: My coworkers always report others if they break any safety rules.

CSSV6: My coworkers always use safety equipment, even when it's not easily available.

2. The 4 items of coworkers' situational safety violations

CRSV1: My coworkers sometimes cut corners if it makes the task easier.

CRSV2: Production pressures mean that coworkers sometimes bend the rules.

CRSV3: Occasionally my coworkers bend the rules when they know it is safe to do so.

CRSV4: When the boss is not around my coworkers can be more flexible with which procedures they follow.

3. The 4 items of perceived social support

PSS1: Management frequently discusses safety issues with the workers.

PSS2: Management can always deal with the safety issues reported by workers in a timely manner.

PSS3: There is frequent communication about safety issues within our workgroup.

PSS4: My coworkers always warn other workers if they break any safety rules.

4. The 4 items of perceived production pressure

PPP1: We often emphasize production goals over safety goals in my workgroup.

PPP2: We are often in such a hurry that safety is temporarily overlooked in my workgroup.

PPP3: Sometimes there is not enough time available for following safety rules and procedures.

PPP4: We take short cuts because we need to get the job done in a timely manner.

PPP5: Short cuts and risk taking are common due to the heavy workload.

PPP6: We often need to finish our work as quick as possible due to heavy work pressure.

5. The 4 items of attitudinal ambivalence towards safety compliance

AASC1: Following safety procedures makes working more difficult.

AASC2: Wearing personal protective equipment bothers my daily work.

AASC3: Wearing personal protective equipment helps me avoid possible damage.

AASC4: Following safety procedures makes me feel safe.

6. The 4 items of safety motivation

SM1: I believe that workplace safety is an important issue.

SM2: I feel that it is worthwhile to put in effort to maintain or improve workplace safety.

SM3: I enjoy working safely on site.

SM4: I feel guilty when I don't work safely.

7. The 6 items of individuals' situational safety violations

ISSV1: I always carry out my work in a safe manner.

ISSV2: I always report all safety-related incidents.

ISSV3: I always wear personal proactive equipment, even when it's inconvenient and uncomfortable.

ISSV4: I never find following safety procedures a hassle.

ISSV5: I always report others if they break any safety rules.

ISSV6: I always use safety equipment, even when it's not easily available.

8. The 4 items of individuals' situational safety violations

IRSV1: I sometimes cut corners if it makes the task easier.

IRSV2: Production pressures mean that I sometimes bend the rules.

IRSV3: Occasionally I bend the rules when I know it is safe to do so.

IRSV4: When my boss is not around I can be more flexible with which procedures I follow.
